# Associations Between Temporomandibular Disorders and Brain Imaging-Derived Phenotypes

**DOI:** 10.1016/j.identj.2024.01.008

**Published:** 2024-02-15

**Authors:** Jun Lin, Dong-Yuan Cao

**Affiliations:** Key Laboratory of Shaanxi Province for Craniofacial Precision Medicine Research, Testing Center of Stomatology, Xi'an Jiaotong University College of Stomatology, Xi'an, Shaanxi, China

**Keywords:** Brain imaging-derived phenotype, Causal association, Mendelian randomisation, Temporomandibular disorders

## Abstract

**Objective:**

Temporomandibular disorders (TMD) affect the temporomandibular joint and associated structures. Despite its prevalence and impact on quality of life, the underlying mechanisms of TMD remain unclear. Magnetic resonance imaging studies suggest brain abnormalities in patients with TMD. However, these lines of evidence are essentially observational and cannot infer a causal relationship. This study employs Mendelian randomisation (MR) to probe causal relationships between TMD and brain changes.

**Methods:**

Genome-wide association study (GWAS) summary statistics for TMD were collected, along with brain imaging-derived phenotypes (IDPs). Instrumental variables were selected from the GWAS summary statistics and used in bidirectional 2-sample MR analyses. The inverse-variance weighted analysis was chosen as the primary method. In addition, false discovery rate (FDR) correction of *P* value was used.

**Results:**

Eleven IDPs related to brain imaging alterations showed significant causal associations with TMD (*P*-FDR < .05), validated through sensitivity analysis. In forward MR, the mean thickness of left caudal middle frontal gyrus (OR, 0.76; 95% CI, 0.67–0.87; *P*-FDR = 1.15  ×  10^−2^) and the volume of right superior frontal gyrus (OR, 1.24; 95% CI, 1.10–1.39; *P*-FDR = 2.26  ×  10^−2^) exerted significant causal effects on TMD. In the reverse MR analysis, TMD exerted a significant causal effect on 9 IDPs, including the mean thickness of the left medial orbitofrontal cortex (*β* = −0.10; 95% CI, −0.13 to −0.08; *P*-FDR = 2.06 × 10^−11^), the volume of the left magnocellular nucleus (*β* = −0.15; 95% CI, −0.22 to −0.09; *P*-FDR = 3.26 × 10^−4^), the mean intensity of the right inferior-lateral ventricle (*β* = −0.09; 95% CI, −0.14 to −0.04; *P*-FDR = 2.23 × 10^−2^), the volume of grey matter in the anterior division of the left superior temporal gyrus (*β* = 0.09; 95% CI,  0.04–0.14; *P*-FDR = 1.69 × 10^−2^), and so forth.

**Conclusions:**

This study provides genetic evidence supporting the bidirectional causal associations between TMD and brain IDPs, shedding light on potential neurobiological mechanisms underlying TMD development and its relationship with brain structure.

## Introduction

Temporomandibular disorders (TMD) are a group of disorders that affect the temporomandibular joint (TMJ), masticatory muscles, and related musculoskeletal structures. These disorders are the primary cause of nondental pain in the orofacial region.^1^^,^^2^ A variety of symptoms span TMD, encompassing localised pain, hindered jaw motion, and TMJ noise during jaw actions and extending to vaguer indicators such as ear discomfort, tinnitus, dizziness, neck ache, and headaches.^3^^,^^4^

TMD affects a significant portion of the adult population, with epidemiologic studies revealing that 15% of adults are affected.^3^ The financial burden of TMD is significant, with annual costs exceeding $100 billion in the US alone.^5^ Despite its widespread impact, the pathophysiology behind TMD remains elusive, demanding more comprehensive research.

Increased evidence from magnetic resonance imaging (MRI) studies has shown widespread structural and functional abnormalities in the TMD patient's brain. Chronic pain in patients with TMD is associated with cortical thickening in specific brain areas, including the primary somatosensory cortex, frontal polar, and ventrolateral prefrontal cortex.^6^ Patients with TMD have decreased gray matter volume in brain regions such as the left anterior cingulate cortex (ACC), right posterior cingulate gyrus, and right anterior insular cortex.^7^ Furthermore, TMD has been associated with structural abnormalities in sensorimotor regions, including decreased cortical thickness in the right sensorimotor cortex and decreased volume in the left putamen.^8^ These findings provide an overview of the structural and functional changes in the brain of patients with TMD.

However, current cross-sectional studies struggle to definitively establish causal connections. Whilst brain changes in TMD patients have been observed, it is challenging to determine whether these alterations are direct outcomes of prolonged pain or are the brain's compensatory mechanisms to alleviate pain. Furthermore, certain brain structural characteristics linked to anxiety, distress, and feelings of helplessness may either predispose individuals to TMD or be reactions to TMD-induced pain, affecting brain anatomy and function.

Mendelian randomisation (MR) is a method to explore causal relationships by leveraging genetic variants as instruments to mitigate confounding biases inherent in observational studies.^9^^,^^10^ This approach is particularly valuable for investigating the causal links between exposures and outcomes. The present study employs MR to probe the bidirectional causal associations between TMD and brain imaging-derived phenotypes (IDPs) using genetic variants associated with TMD and IDPs related to brain imaging alterations to uncover novel insights into the potential neurobiological underpinnings of TMD and its associations with brain structure.

## Methods

### Data sources

#### Summary-level data for brain IDPs

Genome-wide association study (GWAS) summary statistics of brain IDPs were utilised, which were accessed through the Oxford Brain Imaging Genetics (BIG40) web server (https://open. win. ox. ac. uk/ukbiobank/big40/).^11^ The brain imaging data were acquired from the early 2020 participant release involving 40,000 individuals and underwent processing by WIN/FMRIB (Wellcome Centre for Integrative Neuroimaging) on behalf of the UK Biobank.^12^ The processing involved quantile normalisation of each IDP's data vector, which ensured that the data followed a Gaussian distribution, characterised by a mean of zero and a standard deviation of 1. Elaborate descriptions of these neuroimaging metrics and processing methods can be accessed through the online documentation provided by the UK Biobank (https://biobank.ctsu.ox.ac.uk/crystal/crystal/docs/brain_mri.pdf). The GWAS analysis incorporated the complete set of 3929 available IDPs and quality control measures from the UK Biobank, in addition to 6 derived summary connectivity features.^13^ A comprehensive presentation of the GWAS summary statistics on brain IDPs is concisely outlined in Supplementary Table 1.

#### Summary-level data for TMD

Summary statistics pertaining to TMD were obtained from the FinnGen GWAS summary statistics (https://results.finngen.fi/en). FinnGen is a large public-private partnership with the objective of gathering and analysing genomic and health data from 500,000 Finnish biobank participants.^14^ TMD is treated as a binary outcome variable, with 13,282 diagnosed cases identified using specific International Classification of Disease-10 (ICD-10) codes (eg, K07.60, K07.61, K07.62) and a control group comprising 363,995 individuals without a TMD diagnosis (https://risteys.finngen.fi/endpoints/TEMPOROMANDIB_INCLAVO). Detailed information is concisely summarised in Supplementary Table 2, complemented by additional details in the Supplementary Note.

### Genetic correlation analysis

Identifying genetic correlations between complex traits and diseases can provide useful aetiologic insights and help prioritise likely causal relationships.^15^ If traits are causally related and have nonzero heritability, then there should be nonzero genetic correlations.^16^ Thus, genetic correlations were computed utilising the linkage disequilibrium (LD) score regression method (https://github.com/bulik/ldsc).^17^ This method was employed with default parameters and LD scores specific to individuals of European ancestry. A threshold of *P* < .05 for genetic correlation was set to identify suggestive evidence between IDPs and TMD, initiating an analytic process that began with the identification of genetic correlations and then proceeded to MR analysis. This analytics process mitigated the multiple testing challenge^16^^,^^18^ in phenome-wide studies and differentiated between true polygenic signals and bias.^17^

### MR analysis

#### Design

The MR approach employs genetic variants as instrumental variables (IVs) to deduce potential causality between exposure and outcome. The inheritance of genetic variation is random and not affected by environmental factors or disease processes. Therefore, MR is less prone to bias from confounding and reverse causality. For valid MR analysis, 3 key assumptions must be met for the IVs: (1) IVs must associate with the exposure (relevance assumption); (2) IVs must not share common causes with the outcome (independence assumption); and (3) IVs should affect the outcome only through the exposure (exclusion restriction assumption). In other words, valid IVs are genetic variants strongly associated with the exposure but not directly associated with the outcome or confounding factors.

**Fig. 1:**
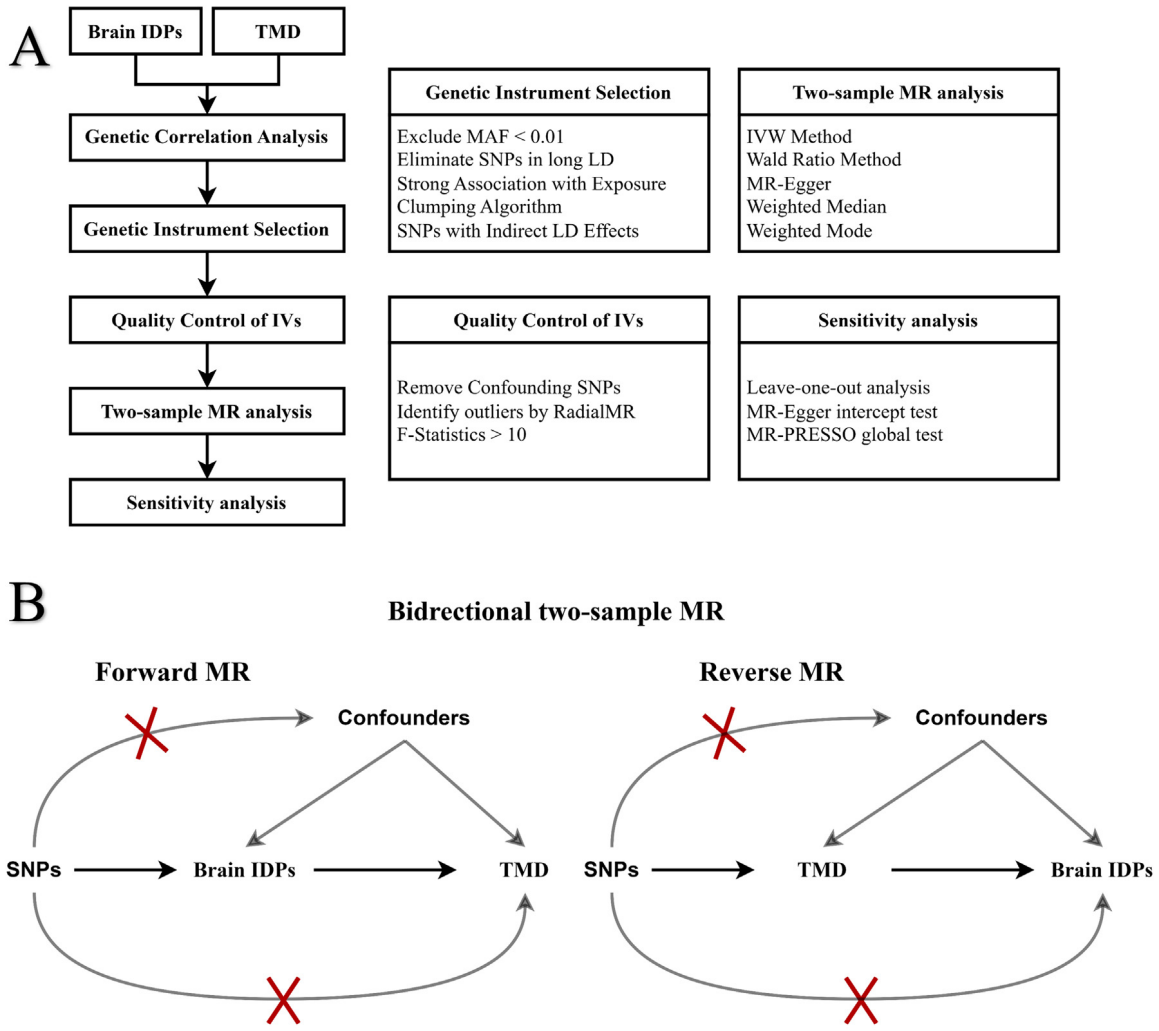
Flowchart of the study (**A**) and schematic representation of the bidirectional Mendelian randomisation (MR) (**B**). In the forward MR analysis, single-nucleotide polymorphisms (SNPs) strongly associated with imaging-derived phenotypes (IDPs), but not with temporomandibular disorders (TMD) or confounding factors, are used as instrumental variables. In the reverse MR analysis, SNPs associated with TMD, but not with IDPs or confounding factors, serve as instrumental variables. The grey lines with red crosses represent the violations of MR assumptions, where the lower line indicates a breach of assumption 2: Instrumental variables (IVs) must not share common causes with the outcome (independence assumption) and the upper line signifies a violation of assumption 3: IVs should affect the outcome only through the exposure (exclusion restriction assumption). IVW, inverse-variance weighted; LD, linkage disequilibrium; MAF, minor allele frequency; MR-PRESSO, Mendelian randomisation pleiotropy residual sum and outlier.

As shown in the overall workflow (Figure 1), MR analysis was used in the present study to explore bidirectional causal links between IDPs and TMD. In the forward MR analysis, brain IDPs were taken as exposures and TMD as outcomes. Conversely, the reverse MR analysis treated TMD as exposures and brain IDPs as outcomes.

#### Genetic instrument selection

For each GWAS summary statistic, several preprocessing steps were undertaken. Variants with minor allele frequencies (MAF) lower than 0.01 were excluded. Furthermore, the elimination of single-nucleotide polymorphisms (SNPs) located within long LD regions was performed to eliminate confounding effects of long-range LD in subsequent research.^19^

After cleaning the above SNPs, IVs for the MR analyses were selected from GWAS summary statistics. Based on the assumption that IVs had a strong association with the exposure variables, SNP IVs were extracted from the GWAS summary statistics of the exposure factor using a genome-wide significance threshold of *P* < 5  ×  10^−8^. For reverse MR analysis, the threshold was adjusted to 5  ×  10^−6^ to encompass more SNPs. A clumping algorithm was utilised to pinpoint independent SNP IVs in order to reduce the impact of LD, using an *r*^2^ threshold of 0.001 and a window size of 1 Mb. Additionally, SNPs exhibiting indirect LD effects (*r*^2^ > 0. 8) were omitted if they demonstrated an association with the outcome variables.

#### Quality control of IVs

To avoid potential confounding, the PhenoScanner V2 database (http://www.phenoscanner.medschl.cam.ac.uk/) was used to remove each instrument SNP and their proxies (*r*^2^ > 0.8) that were significantly associated with confounders between brain IDPs and TMD. Four potential confounders were taken into account: nervousness, anxiety, depression, and tension. These traits were reported by previous studies to affect both brain structures^20^ and TMD.^21^^,^^22^

To enhance the precision and sturdiness of the genetic instruments, heterogeneity tests were performed to identify outliers, which were then adjusted.^23^ Both Cochran Q test and Rucker Q’ test were conducted with the RadialMR package (https://github. com/WSpiller/RadialMR/),^24^ calculating the modified Q and Q’ statistics, respectively. Outliers were discarded based on a nominal significance level of 0.05.

To evaluate weak instrument bias, *F* statistics were performed to measure the strength of IVs, with a threshold below 10 implying greater bias.^25^^,^^26^ The calculation formula is as follows:

$$F=\left(N-K-1\right)\times R^{2}/\left[\left(1-R^{2}\right)\times K\right];\;\;R^{2}=2\times EAF\times \left(1-EAF\right)\;\times \beta^{2}/\left[\left(2\times EAF\times \left(1-EAF\right)\times \beta^{2}+2\times EAF\times \left(1-EAF\right)\times se^{2}\times N\right)\right].$$

*R*^2^ denotes the proportion of variance in the exposure that can be attributed to genetic variants. The sample size is represented by N, while K signifies the number of IVs employed. The genetic effect size derived from the exposure GWAS data is denoted by β, and its corresponding standard error is represented by SE. Additionally, the effect of allele frequency is indicated by EAF.

To ensure the alignment of effect alleles and mitigate potential strand flipping issues,^27^^,^^28^ palindromic SNPs (those with A/T or G/C alleles) with MAF close to 0.5 were removed following the pipeline in the TwoSampleMR R package (https://github. com/MRCIEU/TwoSampleMR).^29^

#### Statistical methods

Two-sample MR analysis was conducted to explore the causal relationships between brain IDPs and TMD. The inverse-variance weighted (IVW) method with multiplicative random effects was selected as the primary analytical approach because of its high efficiency, despite assuming the validity of all SNPs as instrumental variables.^28^ The causal effects were quantified using odds ratio (OR) and β coefficients, both of which were obtained from the IVW method based on a meta-analysis of SNP-specific Wald estimates. Given that brain IDPs were standardised prior to the GWAS,^11^ the OR elucidated the change in the risk of TMD corresponding to 1 SD increment in brain IDPs. Simultaneously, the β coefficient quantified the alteration in brain IDPs, measured in SDs, and this change is attributable to the presence of TMD. When only one genetic instrument was at hand, the Wald ratio method was employed for MR analysis. Considering multiple testing, the Benjamini and Hochberg false discovery rate (FDR) adjustment was used,^30^ with a significance threshold set at 0.05.

For further validation and strengthening of the findings, additional MR methods such as MR-Egger, weighted median, and weighted mode were used. Results were deemed significant in MR analysis if estimates from these supplementary MR methods aligned in direction with the IVW method.

Sensitivity analysis was performed to validate the significance of MR results. A leave-one-out analysis was first executed to gauge whether a solitary SNP significantly affected the causal relationship. Directional pleiotropy was then assessed via the MR-Egger intercept test.^31^ In this test, a nonzero intercept served as an indicator of potential bias in the IVW estimate. The MR pleiotropy residual sum and outlier (MR-PRESSO) global test was utilised to identify the widespread existence of horizontal pleiotropy.^32^

## Results

### Genetic correlations

**Fig. 2:**
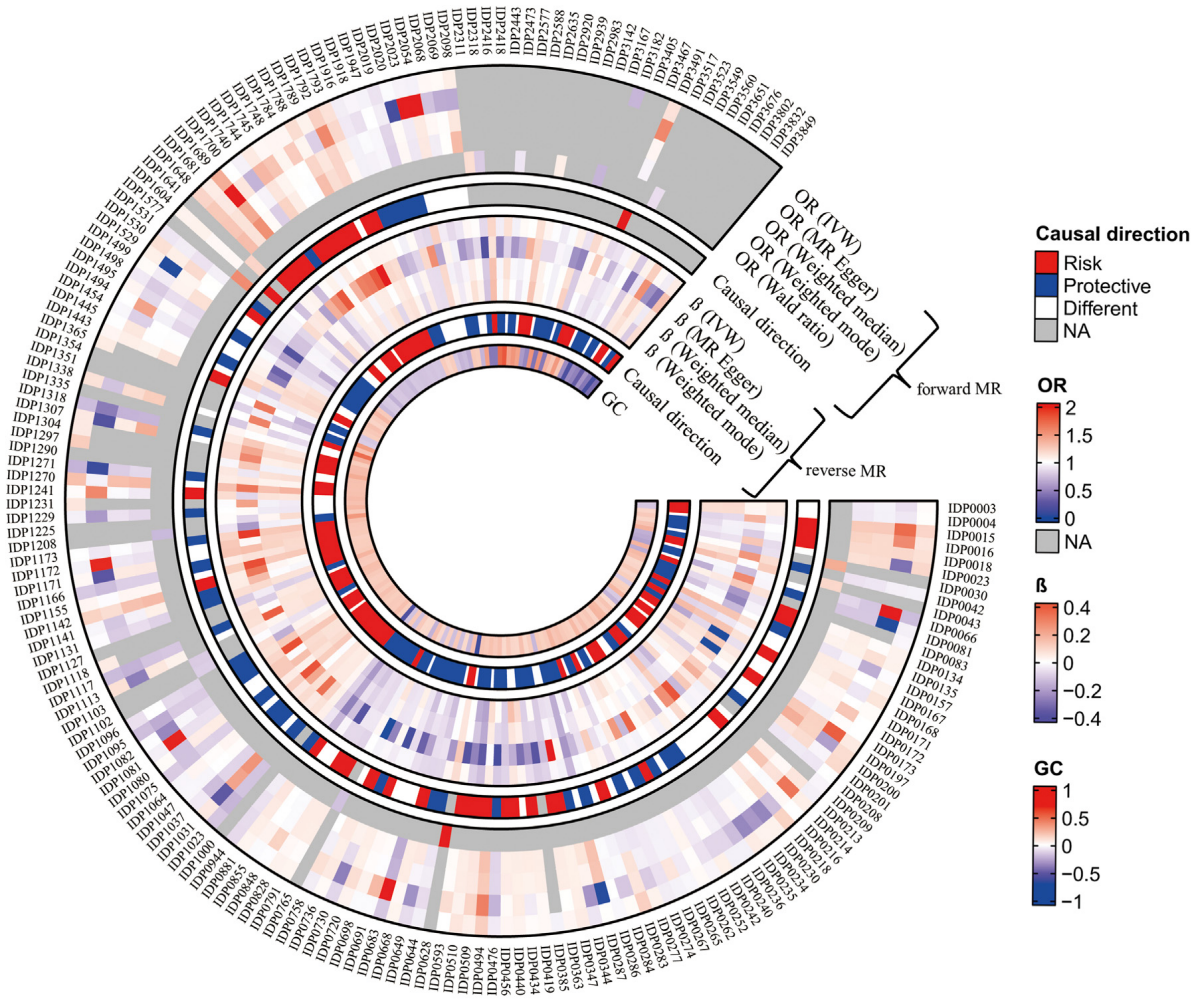
Genetic correlation values and Mendelian randomisation (MR) analysis estimates for imaging-derived phenotype (IDP)–temporomandibular disorders (TMD) pairs with significant genetic correlation. The outermost IDP labels list the brain IDPs that have demonstrated significant genetic correlations with TMD. The odds ratio rings show estimated odds ratios indicating the risk of TMD associated with each IDP, with red for increased risk, blue for decreased risk, and gray for NA. The β rings represent the estimated effect sizes of TMD on IDPs, with red indicating a positive effect and blue a negative effect. The genetic correlation ring illustrates the estimated genetic correlations between TMD and IDPs, with red for positive correlation and blue for negative correlation. The causal direction rings show the estimated directions of different MR methods, where red signifies concordant directions with increased risk, blue signifies concordant directions with decreased risk, and white indicates inconsistent directions across different MR methods. Brackets alongside the ring differentiate between forward MR (outer) and reverse MR (inner) analysis. IVW, inverse-variance weighted; NA, not available.

In the present study, 182 of 3935 brain magnetic resonance IDPs showed significant genetic correlations (*P* <  .05) with TMD. We further investigated the causal associations between these IDPs and TMD using 2-sample MR analysis. Figure 2 presents the genetic correlation values for IDPs-TMD pairs with significant genetic correlations as well as the estimated values for subsequent analyses. The detailed results of genetic correlations are shown in Supplementary Table 3.

### IV selection and quality control

After LD pruning, the IVs associated with potential confounders (Supplementary Table 4) were removed. Outlier IVs (Supplementary Tables 5 and 6) were also removed for subsequent MR analyses. The full lists of IVs used for forward and reverse MR tests are provided in Supplementary Tables 7 and 8. The *F* statistics were calculated to assess instrument strength. For the forward and reverse MR pairs with at least 1 IV, the *F* statistic values were 21.30 to 363.86, suggesting that these SNPs are suitable IVs.

### Causal effects of IDPs on TMD

**Fig. 3:**
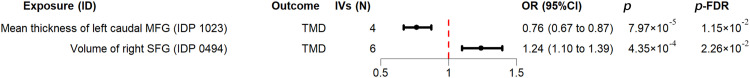
The significant causal results of the forward Mendelian randomisation (MR) analysis. The forest plot shows the causal effect using values obtained by the inverse-variance weighted method. Each point estimate represents the odds ratio (OR), indicating the change in the risk of temporomandibular disorders (TMD) per 1 SD increase in the respective imaging-derived phenotype. The horizontal error bars depict the 95% CI for each estimate. The vertical dashed red line represents the null effect (OR = 1). *P* values are adjusted for false discovery rate. IVs, instrumental variables; MFG, middle frontal gyrus; SFG, superior frontal gyrus.

In the forward MR (IDPs as the exposures), as shown in Figure 3 and Supplementary Table 9, 2 IDPs located in the frontal lobe were causally associated with TMD. A decrease of 1 SD in the mean thickness of the left caudal middle frontal gyrus (MFG) was associated with 24% higher risk of TMD (IVW OR, 0.76; 95% CI, 0.67–0.87; *P-*FDR = 1.15  ×  10^−2^). An increase of 1 SD in the volume of the right superior frontal gyrus (SFG) was associated with 24% higher risk of TMD (IVW OR, 1.24; 95% CI, 1.10–1.39; *P-*FDR = 2.26  ×  10^−2^).

### Causal effects of TMD on IDPs

**Fig. 4:**
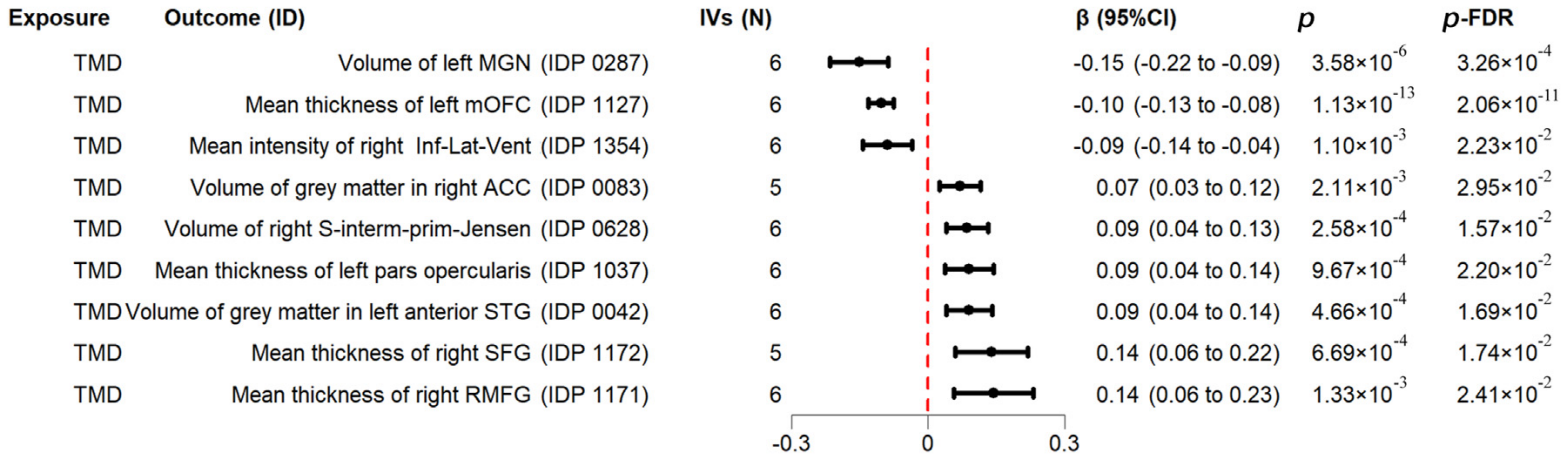
The significant causal results of the reverse Mendelian randomisation (MR) analysis. The forest plot shows the causal effect using values obtained by the inverse-variance weighted method. Each point estimate signifies the change in standardized imaging-derived phenotype attributable to temporomandibular disorders (TMD), with horizontal error bars representing the 95% CI. The vertical dashed red line denotes the null effect (β = 0). *P* values are adjusted for false discovery rate. ACC, anterior cingulate cortex; Inf-Lat-Vent, inferior lateral ventricle; IVs, instrumental variables; MGN, medial geniculate nucleus; mOFC, medial orbitofrontal cortex; RMFG, right middle frontal gyrus; SFG, superior frontal gyrus; S-interm-prim-Jensen, superior intermediate primary Jensen; STG, superior temporal gyrus.

In the reverse MR (TMD as the exposure), as shown in Figure 4 and Supplementary Table 10, the mean thickness of the left medial orbitofrontal cortex (mOFC) (IVW *β*, −0.10; 95% CI , −0.13 to −0.08; *P-*FDR = 2.06 × 10^−11^), the volume of the left medial geniculate nucleus (MGN) (IVW *β*, −0.15; 95% CI, −0.22 to −0.09; *P-*FDR = 3.26 × 10^−4^), and the mean intensity of the inferior-lateral ventricle in the right hemisphere (IVW *β*, −0.09; 95% CI, −0.14 to −0.04; *P-*FDR = 2.23 × 10^−2^) were significantly decreased in patients with TMD.

The volume of the S-intermediate-primary-Jensen region in the right hemisphere (IVW *β*, 0.09; 95% CI, 0.04–0.13; *P-*FDR = 1.57 × 10^−2^), the volume of grey matter in the anterior division of the left superior temporal gyrus (STG) (IVW *β*, 0.09; 95% CI, 0.04–0.14; *P-*FDR = 1.69 × 10^−2^), the mean thickness of the right SFG (IVW *β*, 0.14; 95% CI, 0.06–0.22; *P-*FDR = 1.74 × 10^−2^), the mean thickness of the left pars opercularis region (IVW *β*, 0.09; 95% CI, 0.04–0.14; *P-*FDR = 2.20 × 10^−2^), the mean thickness of the right rostral middle frontal gyrus (RMFG) (IVW *β*, 0.14; 95% CI, 0.06–0.23; *P-*FDR = 2.41 × 10^−2^), and the volume of grey matter in right ACC (IVW *β*, 0.07; 95% CI, 0.03–0.12; *P-*FDR = 2.95 × 10^−2^) were all significantly increased in patients with TMD.

### Sensitivity analysis

To ensure the credibility of our inferred causal relationships through bidirectional MR, sensitivity analysis was performed. First, leave-one-out analysis revealed that the causal estimates were not driven by any single SNP, as illustrated in Supplementary Figures 3 and 4. Second, no evidence of horizontal pleiotropy was found in the MR-PRESSO global test, as supported by the Table. Additionally, the MR-Egger intercepts for all associations were near zero, indicating an absence of significant pleiotropy (Table). In conclusion, these sensitivity analyses validate the reliability of inferred causal effects from both forward and reverse MR findings.

**Table:** Pleiotropy assessment for the significant results of forward and reverse MR analyses.

Exposure	Outcome	MR-Egger intercept			MR-PRESSO global test	
		Egger intercept	SE	*P* value	Observed RSS	*P* value
IDP0494	TMD	−1.85 × 10^−02^	2.15 × 10^−02^	.44	2.18	.92
IDP1023	TMD	1.00 × 10^−02^	2.08 × 10^−02^	.68	1.39	.85
TMD	IDP 0042	−1.49 × 10^−02^	2.09 × 10^−02^	.51	1.94	.93
TMD	IDP 0083	−5.24 × 10^−03^	2.10 × 10^−02^	.82	1.07	.95
TMD	IDP 0287	6.78 × 10^−03^	2.13 × 10^−02^	.77	2.63	.89
TMD	IDP 0628	−4.40 × 10^−03^	2.13 × 10^−02^	.85	1.53	.96
TMD	IDP 1037	9.81 × 10^−04^	2.13 × 10^−02^	.97	1.91	.94
TMD	IDP 1127	−5.86 × 10^−03^	2.13 × 10^−02^	.80	0.50	1.00
TMD	IDP 1171	−1.63 × 10^−03^	2.13 × 10^−02^	.94	4.96	.66
TMD	IDP 1172	1.15 × 10^−02^	3.33 × 10^−02^	.75	2.80	.78
TMD	IDP 1354	−4.71 × 10^−03^	2.13 × 10^−02^	.84	2.04	.93

IDP, imaging-derived phenotypes; MR, Mendelian randomisation; MR-PRESSO, Mendelian randomisation pleiotropy residual sum and outlier; RSS, residual sum of squares; TMD, temporomandibular disorders.

## Discussion

Observational studies suggest a link between IDPs and TMD. However, it is unclear whether this relationship is causal. The present study explored this potential causal relationship using genetic variants obtained from large-scale GWAS datasets and a 2-sample MR approach.

Out of 3935 IDPs examined, 182 displayed genetic correlations with TMD, including variations in structures like the STG and ACC. The Orofacial Pain Prospective Evaluation and Risk Assessment (OPPERA) project identified genes like the 5-Hydroxytryptamine (5-HT, serotonin) receptor 2A (5-HT2A) gene associated with TMD,^33^ and a meta-analysis also highlighted the significance of catechol-*O*-methyltransferase (COMT) gene polymorphisms in TMD.^34^ These discoveries collectively suggest a genetic contribution to TMD. Meanwhile, the 5-HT2A receptor polymorphism is linked to brain structural changes, like the STG.^35^ Another study emphasised the role of COMT genetic variations in brain structures such as the volumes of the temporal lobe and ACC.^36^ These findings may indicate shared genetic factors underlying the genetic correlation between IDPs and TMD.

However, it is important to recognise that genetic correlations alone do not constitute definitive evidence of causality. Such correlations may be affected by genetic confounding or horizontal pleiotropy, and they do not inherently determine the causal direction. Therefore, to further investigate this, bidirectional 2-sample MR analyses were conducted, and 11 IDPs had significant and dependable causal associations with TMD.

The forward MR analysis identified associations between variations in frontal lobe structures and the risk of TMD. More specifically, a decreased mean thickness of left caudal MFG was associated with high TMD susceptibility. The MFG is a critical region in the brain that plays a pivotal role in regulating attention networks.^37^ According to the theory of pain, an attentional bias towards pain contributes to the development and maintenance of chronic pain conditions.^38^ This attentional bias implies that when individuals experience pain or discomfort, their attention is more likely to be drawn to these sensations. Consequently, if an individual has a thinner left caudal MFG, they may be more sensitive to pain or tension in the TMJ. This heightened sensitivity may further exacerbate the symptoms of TMD, as these individuals are more prone to focus their attention on pain or discomfort.

An increase in the volume of right SFG corresponds to a heightened TMD risk. The supplementary motor area (SMA) properly constitutes a significant portion of the superior frontal gyrus, occupying approximately one-third of its overall territory.^39^ The SMA plays a crucial role in planning complex movements and executing deliberate motor actions. Chewing is one such complex movement that requires the intricate coordination of various muscles and structures within the oral cavity. Given the SMA's role and its location within the SFG, unilateral alterations in this brain region may result in unilateral chewing patterns, which create a conducive environment for TMD development.^40^^,^^41^

In the reverse MR analysis, 9 IDPs were identified, indicating that TMD leads to structural changes within the brain regions associated with emotion, pain, cognition, auditory processing, and stress. To be specific, TMD leads to decreased mean thickness of left mOFC. The OFC plays a pivotal role in the emotion circuit,^42^ with its function and structure tied to emotional processing. Previous studies have identified abnormalities in the mOFC associated with various anxiety disorders.^43^ Additionally, a negative correlation has been observed between pain unpleasantness and cortical thickness within the OFC.^6^ Such findings suggest that the enduring pain and psychological stress commonly observed in patients with TMD may induce adaptations in the mOFC.

TMD leads to an increased volume of grey matter in the right ACC. ACC is an important region responsible for encoding the emotional and motivational aspects of pain.^44^ The ACC's augmentation may represent an adaptive neural response, aimed at enhancing its capacity for processing the sustained noxious stimuli inherent to TMD. Given the ACC's robust involvement in pain's affective components and its well-established role in diverse emotional functions, it might serve as a neural bridge linking chronic pain and its ensuing psychological consequences.^45^

TMD leads to increased mean thickness of the right SFG. Notably, the forward MR analysis also indicated an increase in the volume of the same region, which could be a contributing factor to TMD. This volumetric increase may reflect a biological “preparation” or pre-adaptation, whilst the increase in thickness is a direct response or consequence of the presence of TMD. A previous study has shown that there may be a compensatory mechanism in patients with TMD to recruit motor areas such as the SMA to meet elevated demands for motor planning and performance.^46^ Additionally, as a component of the dorsolateral prefrontal cortex, the SFG is canonically associated with higher-order cognitive functions, especially working memory.^47^ Therefore, the increased thickness of the SFG in patient with TMDs may signify the brain's attempt to accommodate the persistent cognitive demands imposed by chronic pain.

There is a reduction in the volume of the left MGN and an increase in the grey matter volume of the left STG. The central auditory pathway processes auditory signals, originating from the cochlear hair cells and proceeding through the auditory nerve, cochlear nucleus, superior olivary complex, inferior colliculus in the midbrain, and the medial geniculate body in the thalamus, ultimately reaching the auditory cortex.^48^ The MGN plays a central role in tinnitus.^49^ Therefore, a decrease in the volume of the left MGN suggests a link between TMD and its auditory-related symptoms, such as tinnitus, which may arise from changes in the central auditory pathway. STG sits at a functional and anatomic interface between lower-level auditory structures and higher-level association areas that support abstract aspects of language.^50^ Observational studies suggest that the temporal lobe might experience volume increases in patients with TMD.^51^ With a diminished MGN volume possibly impeding auditory information transmission and processing, the brain might attempt to compensate by enhancing neural activity and connections in other regions. Given the STG's pivotal role in auditory and language processing, it might be affected by this compensatory mechanism, leading to an increased volume of its grey matter.

TMD leads to increased mean thickness of RMFG in the right hemisphere. A study focussing on college students concluded that there was a significant correlation between TMD and variables such as perceived stress.^52^ Additionally, another investigation found that perceived stress is associated with increased RMFG cortical thickness.^53^ Combining these insights, we hypothesise that perceived stress may mediate the causal effects of TMD on changes in RMFG thickness.

In addition, TMD leads to increased volume of S-interm-prim-Jensen in the right hemisphere, increased mean thickness of pars opercularis in the left hemisphere, and decreased mean intensity of inferior-lateral ventricle in the right hemisphere. The S-interm-prim-Jensen, also known as the first intermediate sulcus, may subdivide the inferior parietal lobule into the supramarginal and angular gyri.^54^ The pars opercularis, along with the pars orbitalis and pars triangularis, form a critical block of Broca's area, which is believed to play a pivotal role in speech production.^55^ The inferior-lateral ventricle is one of the two largest ventricles in the brain. However, few reports have focussed on these alterations in TMD. Further exploration of these changes might pave the way for novel therapeutic strategies for TMD, especially if these alterations are correlated with the severity of symptoms, prognosis, or treatment response in patients.

The present study innovatively adopted MR analysis to investigate the correlation between brain imaging alterations and TMD, enhancing our understanding of the neuropathophysiology underlying the development and experience of TMD. Though our MR analysis cannot replace the comprehensive insights from randomised controlled trials assessing intervention effects, they offer a valuable guide for the design of future costly experiments.

However, our study has several limitations that warrant discussion. First, the study was confined to a European demographic, posing potential issues of ethnic diversity and selection bias that might affect the generalisability of the findings. Future MR studies should prioritise the inclusion of a broader spectrum of populations to foster more comprehensive conclusions. Second, the utilised TMD dataset encompasses numerous subtypes. Given that TMD serves as an umbrella term for a range of painful conditions involving the masticatory muscles, TMJ, and associated structures, there is considerable variability in the disease characteristics amongst patient groups. This variability inhibits our ability to draw firm conclusions about the specific brain alterations associated with distinct TMD types. Last, caution is advised in interpreting the MR estimated effect size, especially when considering its application to clinical interventions. Whilst it is desirable to apply predictive outcomes to inform clinical and health care decisions, it is important to note that the effect size derived from MR studies is based on the study of lifetime exposure factors influenced by exposure risk SNPs, not short-term interventions evaluated in randomised controlled trials. Consequently, it is recommended that these results be applied judiciously in a clinical setting, with a nuanced understanding of the inherent differences in the methodologies employed.

## Conclusions

We performed bidirectional 2-sample MR analyses using large-scale GWAS data to investigate the potential causal relationships between IDPs and TMD. Our findings indicate that alterations in specific brain regions, notably the MFG, MGN, and STG, are associated with TMD. These associations uncover brain regions that may play a role in the development of TMD and also provide insight into the neurobiological basis for various clinical symptoms and comorbidities associated with TMD, such as chronic pain, anxiety, and tinnitus. Ultimately, our study adds to the growing evidence that TMD is interconnected with central pathophysiologic mechanisms, inviting a wider perspective for understanding and addressing this complex condition. These insights may lead to new ways of diagnosing TMD based on brain imaging or even new treatments that target these brain areas to help relieve symptoms.

## Conflict of interest

None disclosed.

## Appendix Supplementary materials

Image, application 1

Image, application 2

## Footnotes

Supplementary material associated with this article can be found, in the online version, at doi:10.1016/j.identj.2024.01.008.
