# Supplementary material for: Associations Between Temporomandibular Disorders and Brain Imaging-Derived Phenotypes
Source: Int Dent J. 2024 Feb 15;74(4):784–93. doi: 10.1016/j.identj.2024.01.008 (PMC11287171; doi:10.1016/j.identj.2024.01.008)
Supplement: Supplementary file 1 [file mmc1.pdf]

Supplementary Figures

Associations between Temporomandibular Disorders and Brain Imaging-Derived Phenotypes

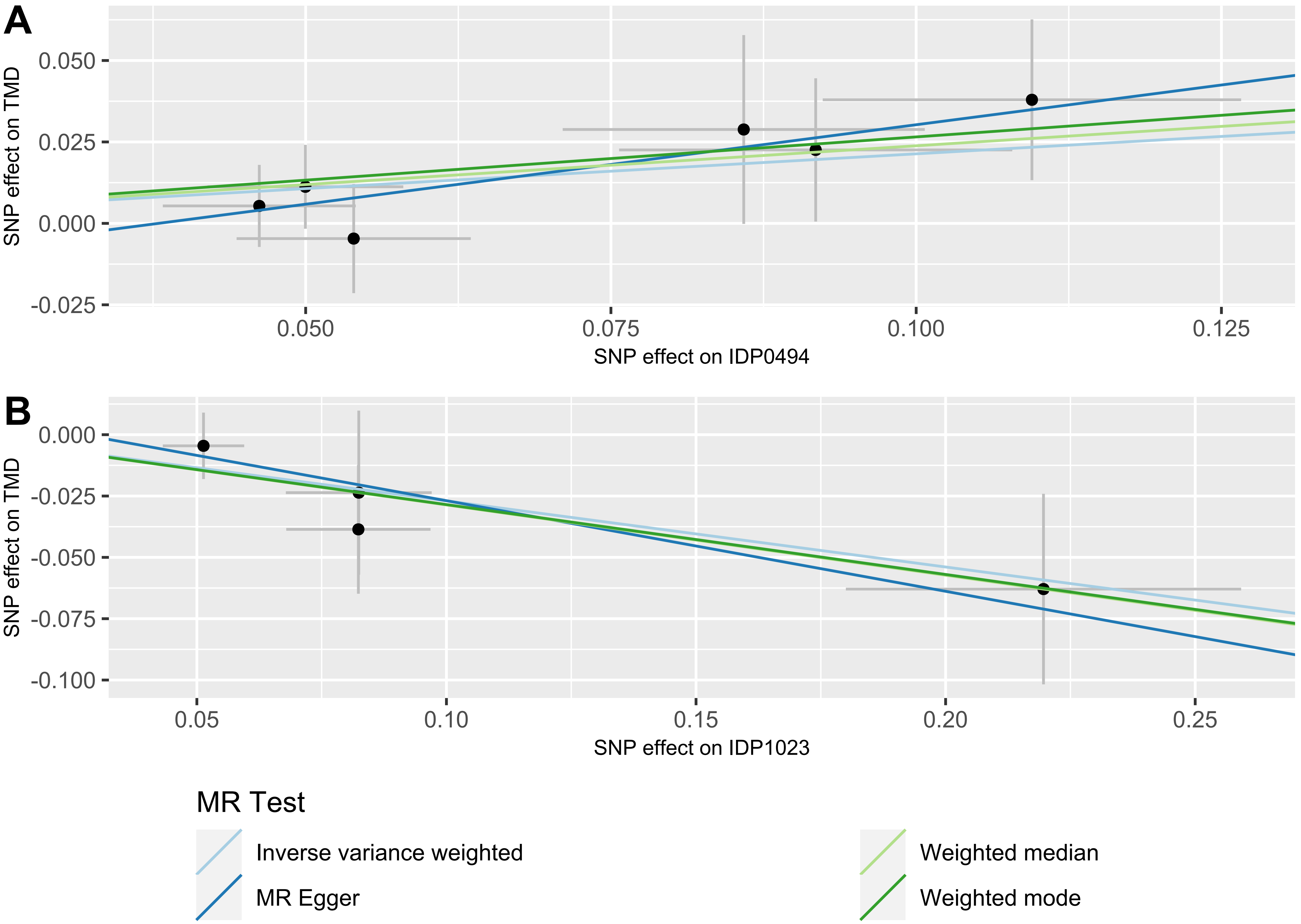
A
SNP effect on TMD
0.050
0.025
0.000
-0.025
0.050
0.075
0.100
0.125
SNP effect on IDP0494
B
SNP effect on TMD
0.000
-0.025
-0.050
-0.075
-0.100
0.05
0.10
0.15
0.20
0.25
SNP effect on IDP1023
MR Test
Inverse variance weighted
MR Egger
Weighted median
Weighted mode

Supplementary Figure 1. The scatter plots of forward MR analysis with significant causal results. (A) The volume of right superior frontal gyrus (IDP0494) as exposure and TMD as outcome. (B) The mean thickness of left caudal middle frontal gyrus (IDP1023) as exposure and TMD as outcome.

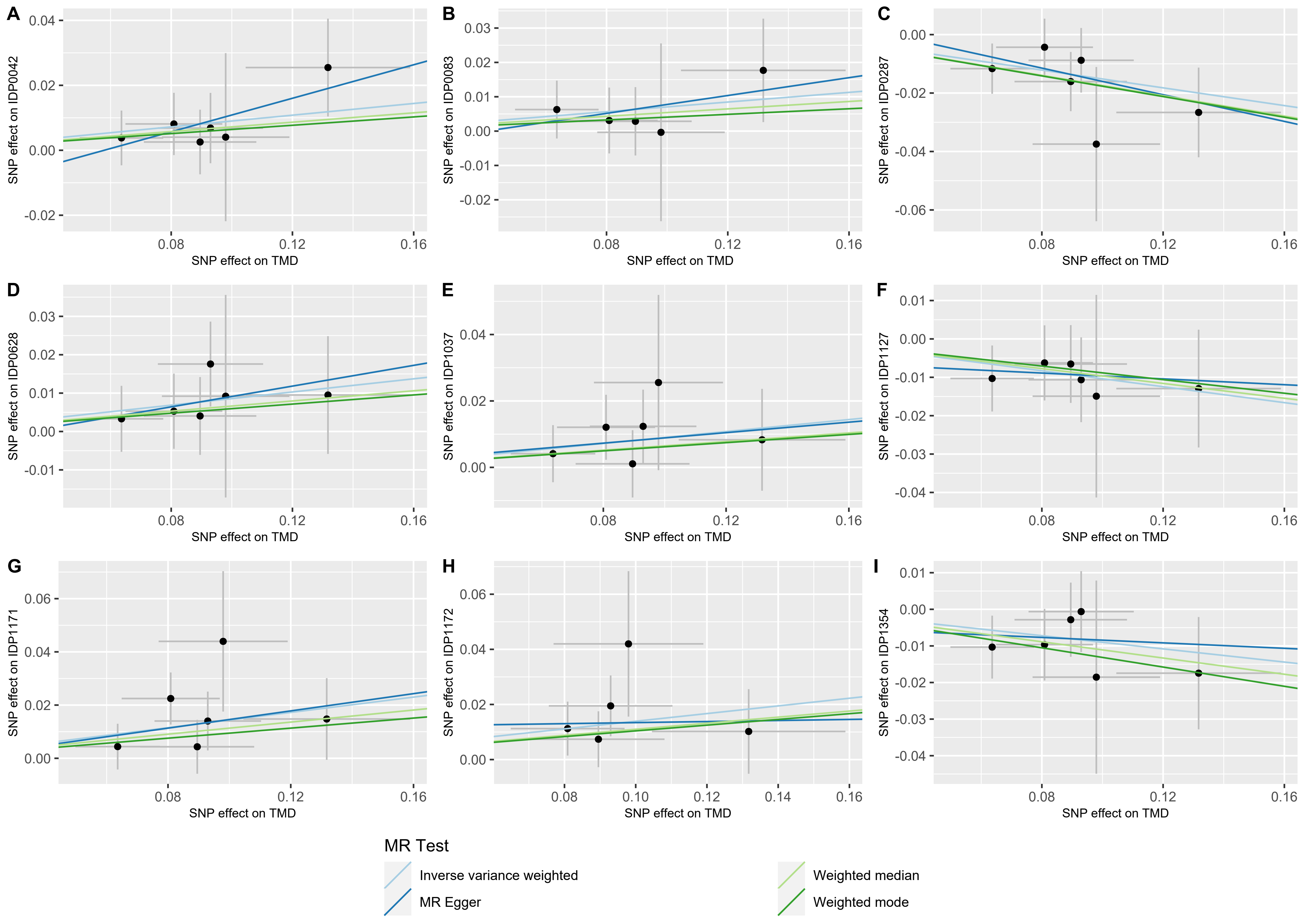
A
B
C
D
E
F
G
H
I
SNP effect on IDP0042
SNP effect on IDP0083
SNP effect on IDP0287
SNP effect on IDP0628
SNP effect on IDP1037
SNP effect on IDP1127
SNP effect on IDP1171
SNP effect on IDP1172
SNP effect on IDP1354
SNP effect on TMD
MR Test
Inverse variance weighted
MR Egger
Weighted median
Weighted mode

Supplementary Figure 2. The scatter plots of reverse MR analysis with significant causal results. (A) TMD as exposure and the volume of grey matter in the anterior division of the left STG (IDP0042) as outcome. (B) TMD as exposure and the volume of grey matter in the anterior division of the right cingulate gyrus (IDP0083) as outcome. (C) TMD as exposure and the volume of the left MGN (IDP0287) as outcome. (D) TMD as exposure and the volume of the S-intermediate-primary-Jensen region in the right hemisphere (IDP0628) as outcome. (E) TMD as exposure and the mean thickness of the left pars opercularis region (IDP1037) as outcome. (F) TMD as exposure and the mean thickness of the left medial orbitofrontal cortex (IDP1127) as outcome. (G) TMD as exposure and the mean thickness of the right rostral middle frontal region (IDP1171) as outcome. (H) TMD as exposure and the mean thickness of the right superior frontal region (IDP1172) as outcome. (I) TMD as exposure and the mean intensity of the inferior-lateral-ventricle in the right hemisphere (IDP1354) as outcome.

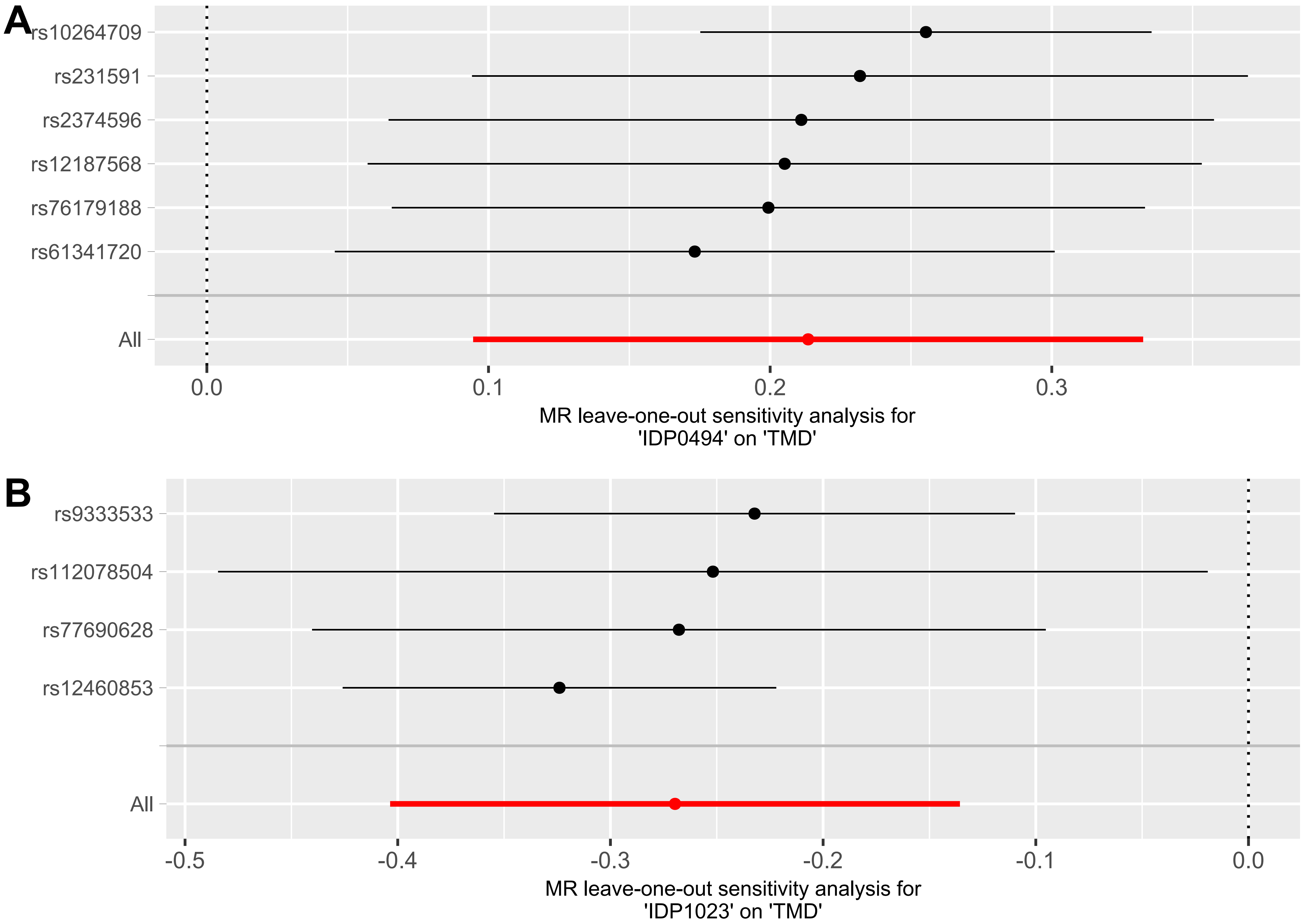
A
rs10264709
rs231591
rs2374596
rs12187568
rs76179188
rs61341720
All
0.0
0.1
0.2
0.3
MR leave-one-out sensitivity analysis for
'IDP0494' on 'TMD'
B
rs9333533
rs112078504
rs77690628
rs12460853
All
-0.5
-0.4
-0.3
-0.2
-0.1
0.0
MR leave-one-out sensitivity analysis for
'IDP1023' on 'TMD'

Supplementary Figure 3. Leave-one-out plots of inverse-variance weighted (IVW) results between IDPs on TMD. (A) The volume of right superior frontal gyrus (IDP0494) as exposure and TMD as outcome. (B) The mean thickness of left caudal middle frontal gyrus (IDP1023) as exposure and TMD as outcome.

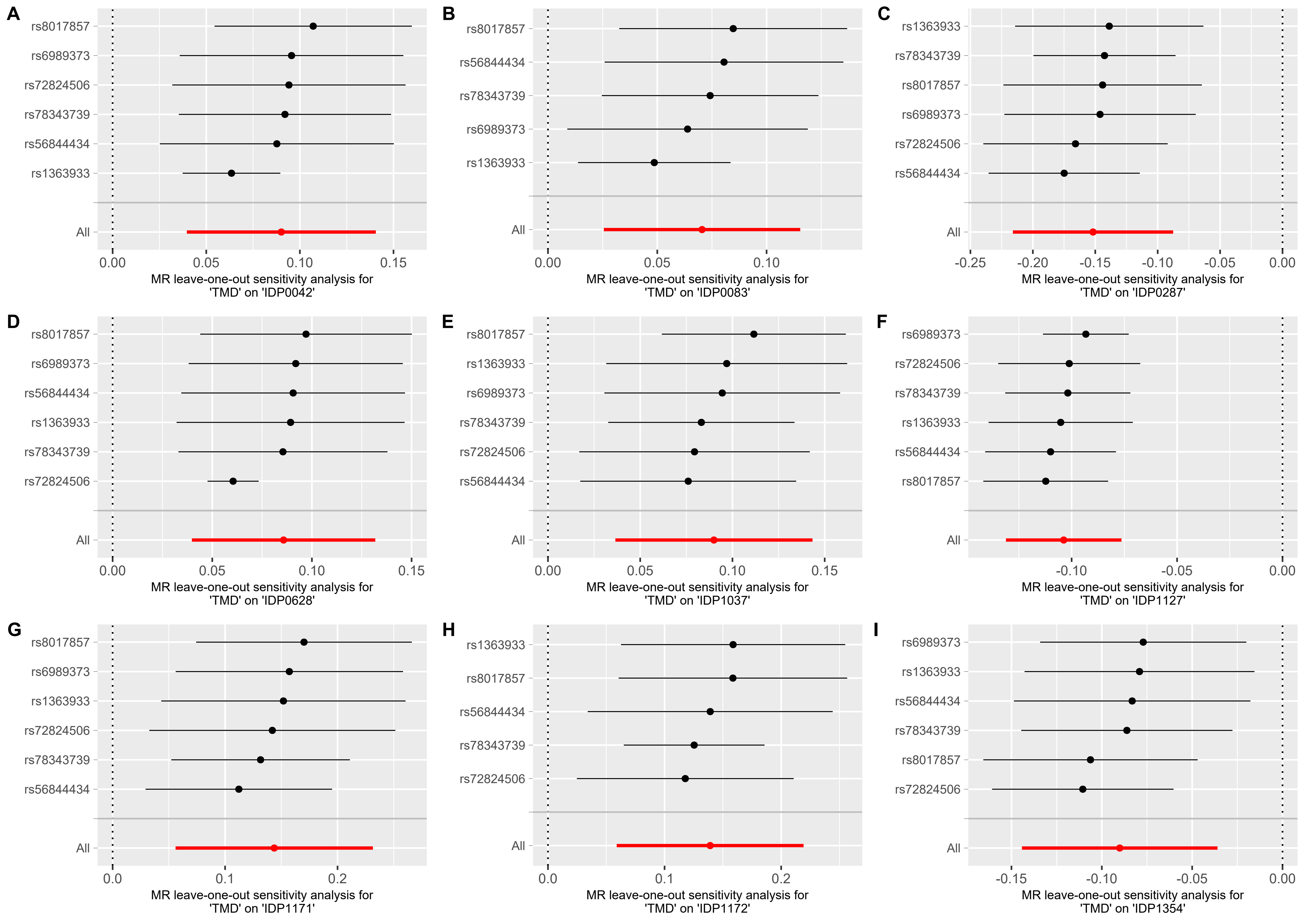
A
rs8017857
rs6989373
rs72824506
rs78343739
rs56844434
rs1363933
All
0.00
0.05
0.10
0.15
MR leave-one-out sensitivity analysis for
'TMD' on 'IDP0042'
B
rs8017857
rs56844434
rs78343739
rs6989373
rs1363933
All
0.00
0.05
0.10
MR leave-one-out sensitivity analysis for
'TMD' on 'IDP0083'
C
rs1363933
rs78343739
rs8017857
rs6989373
rs72824506
rs56844434
All
-0.25
-0.20
-0.15
-0.10
-0.05
0.00
MR leave-one-out sensitivity analysis for
'TMD' on 'IDP0287'
D
rs8017857
rs6989373
rs56844434
rs1363933
rs78343739
rs72824506
All
0.00
0.05
0.10
0.15
MR leave-one-out sensitivity analysis for
'TMD' on 'IDP0628'
E
rs8017857
rs1363933
rs6989373
rs78343739
rs72824506
rs56844434
All
0.00
0.05
0.10
0.15
MR leave-one-out sensitivity analysis for
'TMD' on 'IDP1037'
F
rs6989373
rs72824506
rs78343739
rs1363933
rs56844434
rs8017857
All
-0.10
-0.05
0.00
MR leave-one-out sensitivity analysis for
'TMD' on 'IDP1127'
G
rs8017857
rs6989373
rs1363933
rs72824506
rs78343739
rs56844434
All
0.0
0.1
0.2
MR leave-one-out sensitivity analysis for
'TMD' on 'IDP1171'
H
rs1363933
rs8017857
rs56844434
rs78343739
rs72824506
All
0.0
0.1
0.2
MR leave-one-out sensitivity analysis for
'TMD' on 'IDP1172'
I
rs6989373
rs1363933
rs56844434
rs78343739
rs8017857
rs72824506
All
-0.15
-0.10
-0.05
0.00
MR leave-one-out sensitivity analysis for
'TMD' on 'IDP1354'

Supplementary Figure 4. Leave-one-out plots of IVW results between TMD on IDPs. (A) TMD as exposure and the volume of grey matter in the anterior division of the left STG (IDP0042) as outcome. (B) TMD as exposure and the volume of grey matter in the anterior division of the right cingulate gyrus (IDP0083) as outcome. (C) TMD as exposure and the volume of the left MGN (IDP0287) as outcome. (D) TMD as exposure and the volume of the S-intermediate-primary-Jensen region in the right hemisphere (IDP0628) as outcome. (E) TMD as exposure and the mean thickness of the left pars opercularis region (IDP1037) as outcome. (F) TMD as exposure and the mean thickness of the left medial orbitofrontal cortex (IDP1127) as outcome. (G) TMD as exposure and the mean thickness of the right rostral middle frontal region (IDP1171) as outcome. (H) TMD as exposure and the mean thickness of the right superior frontal region (IDP1172) as outcome. (I) TMD as exposure and the mean intensity of the inferior-lateral-ventricle in the right hemisphere (IDP1354) as outcome.
